# Inclusion of vulnerable groups in health policies: Regional policies on health priorities in Africa

**DOI:** 10.4102/ajod.v2i1.40

**Published:** 2013-01-22

**Authors:** Margie Schneider, Arne Henning Eide, Mutamad Amin, Malcom MacLachlan, Hasheem Mannan

**Affiliations:** 1 Psychology Department, Stellenbosch University, South Africa; 2 Centre for Social Development in Africa, University of Johannesburg, South Africa; 3 SINTEF Technology and Society, Norway; 4 Ahfad University for Women, Omdurman, Sudan; 5 Centre for Rehabilitation Studies, Stellenbosch University, South Africa; 6 Centre for Global Health and School of Psychology, Dublin, Ireland

## Abstract

**Background:**

If access to equitable health care is to be achieved for all, policy documents must mention and address in some detail different needs of groups vulnerable to not accessing such health care. If these needs are not addressed in the policy documents, there is little chance that they will be addressed at the stage of implementation.

**Objectives:**

This paper reports on an analysis of 11 African Union (AU) policy documents to ascertain the frequency and the extent of mention of 13 core concepts in relation to 12 vulnerable groups, with a specific focus on people with disabilities.

**Method:**

The paper applied the EquiFrame analytical framework to the 11 AU policy documents. The 11 documents were analysed in terms of how many times a core concept was mentioned and the extent of information on how the core concept should be addressed at the implementation level. Each core concept mention was further analysed in terms of the vulnerable group in referred to.

**Results:**

The analysis of regional AU policies highlighted the broad nature of the reference made to vulnerable groups, with a lack of detailed specifications of different needs of different groups. This is confirmed in the highest vulnerable group mention being for ‘universal’. The reading of the documents suggests that vulnerable groups are homogeneous in their needs, which is not the case. There is a lack of recognition of different needs of different vulnerable groups in accessing health care.

**Conclusion:**

The need for more information and knowledge on the needs of all vulnerable groups is evident. The current lack of mention and of any detail on how to address needs of vulnerable groups will significantly impair the access to equitable health care for all.

## Introduction

If health policies are to be instruments for realising equity in health, they must specifically document and address the needs of groups vulnerable, or at least potentially vulnerable, in their access to equitable health care services. This paper analyses a series of regional African policy documents to understand the extent to which these documents mention and detail these vulnerability needs, and address the barriers to access to health care.

Acknowledging barriers to people accessing health care has important implications for providing equitable access to health care and ensuring the best possible outcomes in relation to wellbeing and social justice. Disability is one such barrier.^1^ Disability is a common human experience and, in interaction with crucial environmental barriers, can and does limit equitable access to health care. Not accessing health care further exacerbates existing health problems, and puts people with activity limitations at risk for developing new health problems that could easily have been prevented. For example, if a person in a wheelchair is unable to access the health service they will not visit the facility when having bronchitis, leading to further complications if not treated or resolved. Disability is not the only reason why people are limited in their access to equitable health care, but is the focus of this paper. Other groups that are often limited in their access to equitable health care include people living in poverty, women-headed households, orphans and other children with special needs, ethnic minorities, youth, displaced people and people with chronic illnesses (Dixon Woods *et al*. 2005; Goudge *et al*. 2009; Makwiza *et al*. 2009; Panter-Brick 2002; Perry *et al*. 2007; Reichard, Sacco & Turnbull 2004; Ridde 2008).

A number of health care priorities have been identified for the African continent by the African Union (AU), including tuberculosis, malaria, HIV and/or AIDS, other infectious diseases, and sexual and reproductive health. Accordingly, the AU has developed a number of policies, strategies and plans of action to guide AU member states in managing these health priorities at a national level. In addition to these health-focused documents, there are series of policy documents for sectors of the population seen as being vulnerable and at risk for not accessing the necessary health care services. These include documents related to women, youth, elderly people, children and people with disabilities, as shown in the list of documents reviewed and provided below. This reflects recognition of the potential vulnerability of certain groups within populations.

Equitable access to health care is more likely when issues from both health-focused and vulnerability-focused policy documents are integrated into single policy documents with integrated implementation plans. This ensures that issues of vulnerability are mainstreamed and included in all policies rather than being treated as a special case and as a separate and often ‘afterthought’ issue. We argue that mainstreaming at the level of policy documents is a step in the direction of ensuring equitable access to health care truly for all.

Buse *et al*. (2007:1) describe how ‘[p]olicy analysis can contribute to meeting health objectives and untangling the complex forces of power and process that underpin change’. Thus, to meet the health objectives of equitable access to health care by vulnerable groups, we need to understand the extent to which their needs are integrated into health related policies, as a step towards understanding the ‘complex forces’ that influence this integration and lead (or not) to equitable access to health care. If vulnerable groups and their specific needs are not visible or are seen as a separate issue not for inclusion in a ‘mainstream’ policy, their needs are not likely to be addressed and integrated into such ‘mainstream’ policies, and even less at the implementation and budget allocation stages. Thus, it is of interest to determine the extent to which such groups are included in policy documents in order to advocate for such inclusion if required.

The EquitAble project^2^ has as one of its aims to determine the impact of a range of vulnerability factors in equitable access to health care with a specific focus on the impact of disability. The first stage of the EquitAble project comprised an analysis of policy documents to determine whether the identified vulnerability factors are integrated into existing policies on health care provision. The national policies of four African countries (Sudan, Namibia, Malawi and South Africa), regional policies developed by the AU for its member states, and a range of international policies were analysed. The policies considered are those related to health care service provision and managing specific health care priorities in the African region. This paper reports on the analysis of the regional policies developed by the AU for its member states. The analysis of individual country policies are presented in a separate paper (Mannan *et al*. 2012).

### Equitable access to health care and EquiFrame

The United Nations Committee on Economic, Social and Cultural Rights (2000:1) describes health as ‘a fundamental human right indispensable for the exercise of other human rights’ and sets out the four intersecting elements ensuring equitable access to health care services: accessibility, availability, acceptability and quality. This is the definition of equitable health care access adopted by the EquitAble project and used in the development of the policy analysis framework called EquiFrame (Mannan *et al*. 2011; MacLachlan *et al*. 2011). EquiFrame (Mannan *et al*. 2011) is a policy analysis framework that uses a set of core concepts that, if mentioned in a policy, ‘informs the analyst concerning what the policy is [and] what it is intended to accomplish’ (Stowe & Turnbull 2001:206). The core concepts were extracted from work done specifically on disability policy analysis within a human rights framework (Reichard *et al*. 2004; Stowe & Turnbull 2001; Turnbull, Beegle & Stowe 2001; Turnbull & Stowe 2001). This yielded 18 core concepts, to which were added three identified by the United Nations Committee on Economic, Social and Cultural Rights (2000) as being important components of determinants of equitable access to health care. These additional three core concepts were accountability, quality and access, including equitable access to health care as a basic human right (Gilson *et al*. 2008; Russel & Gilson 2006). While all 21 core concepts were considered in the analysis (Table 1), this paper focuses on 13 core concepts (marked with an asterisk*) that were identified in the regional policies beyond an occasional mention in one or two policy documents.^3^

**TABLE 1:** *EquiFrame*: Key questions and key language of core concepts.

Number	Core concept	Definition/Key question
1.	Non-discrimination	Does the policy support the rights of vulnerable groups with equal opportunity in receiving health care?
2.	Individualised services	Does the policy support the rights of vulnerable groups with individually tailored services to meet their needs and choices?
3.	Entitlement	Does the policy indicate how vulnerable groups may qualify for specific benefits relevant to them?

Vulnerable groups are ‘social groups who experience limited resources and consequent high relative risk for morbidity and premature mortality’ (Flaskerud & Winslow 1998:69). The focus of the EquiFrame (Mannan *et al*. 2011) is vulnerability, specifically in relation to accessing health care. Vulnerable groups included in the EquitAble analysis were women, children, elderly people, ethnic minorities, displaced people, people suffering from some illnesses and people with disabilities. Other groups, such as sexual minorities, can also be considered vulnerable but were not included in the analysis. The focus of the analysis was on developing and using a policy analysis tool rather than being fully exhaustive of all vulnerable groups.

Vulnerability is a complex process whereby individual characteristics of a person or group of people put that person at risk of not accessing, for example, health care. The risk factor in itself does not cause limited access, but rather the interaction of the factor with a range of other factors external to the person results in an outcome of lack of access. Thus, having an impairment does not in itself determine access to health care, but the interaction of having an impairment, such as loss of lower limb mobility, long distances to the health facility, poor or expensive transport, and inaccessible buildings all contribute to an outcome of inequitable access to health care for that person. The role of policies is to ensure that these external factors are set out and elucidated in order to ensure effective implementation. This needs to be done for the different needs of different vulnerable groups.

Mannan *et al*. (2011) identified the 12 key vulnerable groups from a review of the literature. An additional category ‘universal’, was included to capture information clearly referring to vulnerable groups but not sufficiently specific in its mention to allocate to one of the other 12 groups (Table 2). Two of the groups were omitted from this regional analysis – ‘increased relative risk for morbidity’ and ‘mother and child mortality’ – as these are more complex and require access to health care to be given a diagnosis of a health condition for the first, or a range of factors outside of a narrow scope of health care for the second.

**TABLE 2:** List and definition of vulnerable groups.

Number	Vulnerable group	Attributes or definitions
1.	Limited resources	Referring to poor people or people living in poverty.
2.	Increased relative risk for morbidity	Referring to people with one of the top 10 illnesses, identified by WHO, as occurring within the relevant country.
3.	Mother child mortality	Referring to factors affecting maternal and child health (Children 0−5 years).
4.	Women-headed household	Referring to households headed by a woman.
5.	Children (with special needs)	Referring to children marginalised by special contexts, such as orphans or children on the street.
6.	Aged	Referring to older age.
7.	Youth	Referring to younger age without identifying gender.
8.	Ethnic minorities	Referring to non-majority groups in terms of culture, race or ethnic identity.
9.	Displaced populations	Referring to people who, because of civil unrest or unsustainable livelihoods, have been displaced from their previous residence.
10.	Living away from services	Referring to people living far from health services, either in time or distance.
11.	Suffering from chronic illness	Referring to people who have an illness which requires continuing need for care.
12.	People with disability	Referring to persons with disabilities, including physical, sensory, intellectual or mental health conditions, and including synonyms of disability.
13	Universal	Making general reference to vulnerable groups without being specific enough to be categorised under any one group.

WHO, World Health Organization.

### Aim and objectives

The aim of this paper is to review the extent to which policies^4^ addressing regional health priorities document the needs of vulnerable groups, as reflected in the core concepts. The underlying assumption is that their inclusion within policies will increase the likelihood of effective and integrated management on the ground. This assumption is not tested in this paper but is addressed in later papers from the EquitAble project that look at the realised access to health care services in four African countries. The focus is on a review of the actual policy documents only and not of the policy development process or its implementation.

The objectives of the paper are to:

1. Identify relevant health policies for the region of Africa developed by the African Union.
2. Review these policy documents in relation to the frequency and extent of mention of the identified vulnerable groups and core concepts.

## Methodology

The research design is exploratory in nature and consists of reviews and analysis of key health-focused policies using the EquiFrame matrix of 13 of the 21 core concepts and 13 vulnerable groups, including the ‘universal’ category.

The EquiFrame matrix provides a detailed analysis of which core concept is mentioned for which vulnerable groups and the nature of the mention, as set out in Appendix 1, as an example. The nature of the mention was rated according to a scale, as set out below. The higher the rating the more comprehensive and detailed the nature of the mention. Ratings of 3 and 4 would be required in order to address the different needs of different vulnerable groups:

0 = Concept not mentioned at all

1 = Concept only mentioned but not developed (incidental)

2 = Concept mentioned and explained (notable)

3 = Specific policy actions identified to address concept (central)

4 = Intention to monitor concept expressed (fundamental)

N/A = Not relevant

Regional policies refer to policies developed within Africa and by the main African regional body, the AU. The AU is a body that brings together African countries and develops policies on issues related to specific African continent needs and problems.

The documents analysed for this report are recommendations from the AU Assembly to member countries, with the aim of providing guidelines for member states to develop their own national level policies. The documents reviewed include charters, protocols, declarations, policy frameworks, strategies and a plan of action. These documents deal with continent-wide issues of importance in relation to health, such as HIV and/or AIDS, Malaria, TB, and sexual and reproductive health services.

These documents are usually prepared by experts who present them to the council of ministers from member states (in this case Ministers of Health) to review and adopt accordingly. For the purpose of this report, the documents reviewed will be referred to as policy documents.

### Criteria for selection of regional policies

There were a number of criteria applied to the selection of the policy documents. The exploratory nature of the research allowed us to select key policies to use in the exploration rather than being exhaustive. In addition the scope of the policy had to be health or health care services, be a current document (even if dated from 10 to 15 years ago), and be regionally focused in their scope. The documents were obtained from a review of the health and health related sections of the AU website.

The following 11 documents were selected and analysed. All are available from the AU website (AU n.d.):

1. African charter on human and people’s rights – Banjul, Organisation for African Unity (OAU) document, 1981.
2. African charter on the rights and welfare of the child – 1990, entered into force 1999 – OAU.
3. Protocol to the African charter on human and people’s rights on the rights of women in Africa. – 1995 adopted in 2003.
4. Africa summit on roll back Malaria – 25 April 2000, Abuja – OAU document.
5. Abuja declaration on HIV and/or AIDS, TB and other related infections diseases – OAU, April 2001.
6. Pan-African forum for children – OAU document – May 2001.
7. Maputo declaration on HIV/AIDS, tuberculosis, Malaria and other related infectious diseases – July 2003 – AU document.
8. Draft continental policy framework for the promotion of sexual and reproductive health and rights in Africa. – AU, October 2005.
9. Plan of action on sexual and reproductive health and rights (Maputo Plan of Action) 2007–2010 – AU document, September 2006.
10. AU youth charter – AU, July 2006.
11. Africa health strategy: 2007–2015 – AU document, April 2007.

### Analytical process

The policies were analysed by two researchers independently, followed by a comparison of the ratings used. Differences were discussed and a consensus reached on what rating to use. The core concepts were rated under the vulnerable group referred to in the policy. If no mention was made of any particular vulnerable group, but the reference to these groups was clear (e.g. ‘all vulnerable groups’), the rating was placed under ‘universal’. Thus each policy has a resulting completed matrix. (See Appendix 3 for an example of such an analysis). The mentioning of a core concept was rated from 1 to 4 depending on the nature of the mention as set out above. The analysis consisted of scanning the written text for mentions of core concepts and vulnerable groups. Terms that were contained in the definition of the concepts of groups were noted as mentions, and, in addition, different terms but that had the same meaning. The focus was on the meaning of the core concept or vulnerable groups rather than strict use of the actual term. For example, ‘limited resources’ and ‘poor’ were noted as being about the same vulnerable group.

Once the policy was analysed and the ratings set out on the matrix, the 11 policies were summarised with respect to the number of core concepts mentioned and for which vulnerable group these were mentioned, as set out in Appendix 2.

If a policy was a general one, those sections that made reference to health and health care and the preamble and guiding principles were identified within the policy and analysed in detail. For example, if a document dealt with issues ranging from health, education and employment, then only the preamble and the specific health clauses were considered for analysis. The language used in the policy was the basis for the analysis.

If there was more than one occurrence of a core concept for a particular vulnerable group, all instances were recorded under the ‘number column of the matrix’ but only the highest rating was included under the relevant vulnerable group. The analysis presented below focuses on the number of times a core concept was mentioned, followed by a separate description of the rating.

## Ethical considerations

As this was a document review no ethical clearance was required.

## Results and discussion

The analysis confirmed that the regional documents provide, in general, broad guidelines for individual countries to develop their own national level policies and guidelines. Very few implementation plans or monitoring and evaluation suggestions were made. While guidelines for these latter components should be provided in regional policies, the detail would be the domain of the individual country policies.

### The vulnerable groups

The 11 documents together mention all 13 vulnerable groups (VGs) (Table 3). However, the most commonly mentioned VGs across all policies are ‘universal’ (in 10 policies) and ‘youth’ (7 policies). The other VGs were only mentioned 1−3 times across the 11 policies. The least mentioned VGs (only mentioned in 1 policy) were ‘limited resources’, ‘women-headed households’ and ‘displaced populations’.

**TABLE 3:** Number of mentions for each vulnerable group in 11 regional policies.

Vulnerable groups	Number of times mentioned	Number of policies in which VG is mentioned
Limited resources	0	0
Women-headed households	0	0
Ethnic minorities	0	0
Elderly	4	3
Displaced populations	5	2
Living far from services	5	3
With disabilities (including youth with disabilities)	7	4
Children with special needs	9	4
With chronic illness	10	3
Youth (without disabilities)	40	7
Universal	148	10

VG, vulnerable groups.

The high rate of mention for youth refers to youth generally and does not include youth with disabilities, who were counted under ‘with disabilities’. This number is explained in part by the inclusion of the AU Youth Charter. When looking at youth specifically, the analysis shows that 25 mentions of this vulnerable group were spread across 6 policies, excluding the Youth Charter, and 14 mentions in the Youth Charter in relation to health care. This suggests that issues of youth access to health care are reasonably well addressed in most policies and that the Youth Charter does address health care access as one of its concerns. The Youth Charter includes three mentions of youth with disabilities (counted under ‘with disabilities’), and one of ‘living far from services’. This finding is congruent with the fact that policies that address specific groups, such as youth, will address needs of that group and will not address needs of some of the other vulnerable groups.

The large number of ‘universal’ mentions (relative to the other 12 specific vulnerable groups) highlights the nature of the policy content, which remains broad and lacking in specificity in relation to those sectors of the population who may struggle to access health care. For example, a policy would mention ‘all vulnerable groups’ but not specify which these would include. Whilst it is acknowledged that this is a step in the right direction, there remains a gap as to how the needs of such groups are to be met within the scope of a policy. It sounds more like a token mention of vulnerable groups than a serious consideration of the aspects to be addressed in order to meet their needs. Furthermore, the lack of specificity obscures the different needs of different groups.

### Core concepts

The frequency of occurrence of each of the 13 core concepts in the 11 documents is presented below (Table 4). The numbers in the first column are those allocated to the core concepts as set out in Table 1 above. The last column gives the number of times each core concept was mentioned in total across all the policies.

**TABLE 4:** Number of core concepts mentioned in 11 regional policies (ranked by total number of CC mentioned).

Core concept	Number of policies mentioning core concept (out of 11 policies)	Total number of core concept mentions
1. Protection from harm	6	17
2. Prevention	9	23
3. Autonomy	0	0
4. Privacy	0	0
5. Non-discrimination	6	22
6. Cultural responsiveness	3	8
7. Coordination of services	6	32
8. Capacity building	7	42
9. Individualised service	6	22
10. Accountability	3	12
11. Quality	6	14
12. Access	6	28
13. Efficiency	2	15

CC, core concept.

Across the 11 policies, ‘capacity building’ was the most frequently mentioned core concept (42) followed by ‘coordination of services’. Apart from ‘autonomy’ and ‘privacy’, which were never mentioned, ‘cultural responsiveness’ was mentioned least. These results reflect the scope of the policies as pushing for capacity building of health care providers and ensuring that services are provided in a coordinated manner. ‘Access’ is mentioned 28 times across 6 policies. However, as shown in the more detailed analysis on ‘access’ below, the majority of these mentions are about a broad notion of ‘equitable access to health care’ in some form or another. Making services physically accessible and providing information in an accessible format are not reflected as being important components of health care provision, as they are not mentioned specifically. This trend is counteracted to some extent by the relatively frequent mention of ‘non-discrimination’ and ‘individualised services’. Both are mentioned in six policies. Mentions of these two core concepts remain general (e.g. ‘no discrimination for anyone’ and ‘services relevant for all’) rather than specifying different needs of different vulnerable groups, especially disabled people.

These results all reflect the broad nature of these regional documents and their emphasis on building strong health care services that provide equitable access for all but with little unpacking of what equitable access entails for individual vulnerable groups or what ‘for all’ really means.

#### Rating of mentioned core concepts

The majority of core concepts were only mentioned (rating 1) or mentioned and explained (rating 2). Very few were mentioned together with a specific policy action identified to address the core concept (rating 3). None of the mentioned core concepts included any discussion on intentions to monitor the core concept addressed (rating 4). Only four policies had ratings of ‘3’ identifying any policy action. These were the Pan African Forum for Children (May 2001), the draft continental policy framework for promotion of sexual and reproductive health rights in Africa (October 2005), AU Youth Charter (July 2006) and the African health strategy (2007).

Core concepts that were coded as having a specific policy action were ‘protection from harm’, ‘prevention’, ‘non-discrimination’, ‘capacity building’, ‘individualised services’, ‘accountability’, ‘quality’ and ‘access’.

#### Analysis of access references

The notion of accessibility is one of the four components that ensure equitable access to health care, as discussed at the start of this paper, with the other three being acceptability, availability and quality. Accessibility is crucial in facilitating equalisation of opportunities for people with disabilities, making it important to look at this aspect in more detail. The further analysis of access only is driven largely by the high number of mentions of this core concept (28 mentions across 11 policies) relative to the other three. The relevant core concept is ‘access’.

There are different components to accessibility – physical, financial and information. The references to ‘access’ in the 11 documents were reviewed in relation to these three components. An additional type of mention is a more general point about ‘access to services’ which is common in the regional documents. The documents frequently make reference to the need for access to health care services but without any further specifications of what this entails.

In total there were 28 mentions of ‘access’, ranging from zero to nine mentions per document (Table 5). The more general category of ‘equitable access to services’ is the largest of the mentioned components, confirming the broad-stroke nature of the regional policies. While this category does not specifically refer to any of the three components – physical, financial and information access – they are implicit in the phrase ‘access to universal and equitable health care services’ or variations on that phrase. However, given the discussion above, it may be insufficient for these to be mentioned implicitly, and it would be preferable for these policies to make more explicit what is required in order to ensure access to universal and equitable health.

**TABLE 5:** Analysis of access mentions in eleven regional documents.

Regional Policy Document		Number of mentions	Content of mentions
1.	African charter on human and people’s rights – Banjul, OAU document 1981	0	**–**
2.	Protocol to the African charter on human and people’s rights on the rights of women in Africa – 1995, adopted in 2003	1	Access to health care services 1
3.	Maputo declaration on HIV/AIDS, Tuberculosis, Malaria and other related infections diseases – July 2003 – AU document	1	Financial (affordable drugs) 1
4.	African charter on the rights and welfare of the child – 1990, entered into force 1999 – OAU	2	Physical (buildings) 1 Information (training) 1
5.	Abuja declaration on HIV/AIDS, TB and other related infections diseases – OAU – April 2001	3	Access to health care services 1 Financial (Affordable drugs and vaccines) 2
6.	Africa summit on roll back malaria – 25 April 2000, Abuja – OAU document	3	Access to health care services 2 Financial access (resources required) 1
7.	Pan-African forum for children – OAU document – May 2001	3	Information 1 Access to health care services 1 Financial (Affordable drugs) 1
8.	AU Youth Charter – AU July 2006	6	Access to health care services (equitable access; programmes to address health pandemics; timely access to health care) 4 Information (youth friendly services) 1 Physical (infrastructure and mobility for access) 1
9.	Africa health strategy: 2007 – 2015 – AU document, April 2007	8	Services (essential health care) 4 Financial (essential drugs; cost; exempt from taxes) 3 Physical (distance and time to reach services) 1
10.	Draft continental policy framework for the promotion of sexual and reproductive health and rights in Africa – AU, October 2005	9	Access to health care services (universal access) 6 Financial (affordable supply and use of ARVs; increasing resources for services) 2 (information for youth) 1
11.	Plan of action on sexual and reproductive health and rights (Maputo Plan of Action) 2007–2010 – AU document, September 2006	9	Access to health care services (universal access) 7 Financial (basic medicines available; quality and affordable services) 2

AU, African Union; OAU, Organisation for African Unity; ARV, Antiretroviral drugs.

The issue of affordable drugs or access to drugs have been coded under ‘financial’ because of its overt reference to financial aspects of this reference – affordable.

The lack of further specifications in the ‘access to universal and equitable health care’ may lead to lack of implementation in a context where there are many implementation issues to be considered. This lack, furthermore, may reflect poor or no knowledge of the required specifications and/or the human rights implications of not addressing specific needs of different vulnerable groups. Given the argument that policy documents must make clear reference to the needs of vulnerable groups and ways to meet these through implementation as one of the components of ensuring universal and equitable access to health, it is worrying that the regional policy documents fail to provide more detailed specifications.

## Conclusions and recommendations

The first objective of this paper was to identify relevant AU policies for the African region. We identified 11 policies that are current in their application, ranging from ones focused on specific groups (e.g. Youth Charter) through to one focused on specific issues (e.g. Draft continental policy framework for the promotion of sexual and reproductive health and rights in Africa). These policies are not exhaustive by any means, but provide a way to start the process of policy analysis proposed in this paper. Given the usefulness of this analysis in highlighting gaps in these 11 policies, the recommendation is to continue this type of analysis on a wider range of health and other policies.

The second objective of reviewing these policies in relation to core concepts and vulnerable groups showed that there are important gaps in the way the core concepts and vulnerable groups are addressed in these policies. The very general nature of reference to vulnerable groups and the limited mention of core concepts suggests that there is limited scope for guiding countries in developing their national policies.

The analysis of regional AU policies highlighted the broad nature of the reference made to vulnerable groups, with a lack of detailed specifications of different needs of different groups. This is confirmed in the highest vulnerable group mention being for ‘universal’. The reading of the documents suggests that vulnerable groups are homogeneous in their needs, which is not the case. The needs of a people living far from a health facility are not the same as those for a deaf person attending a clinic. The first requires adequate transport while the second requires an accessible form of communication, such as sign language. This type of nuance is lost in general mentions of vulnerable groups.

The regional nature of the policies may explain some of the lack of detail concerning relevant policy actions and intention to monitor, as this would be more suitable in national level policies. However, one would like to see regional policies provide guidelines on these at least. In particular, the number of ratings of 3 and 4 should be increased. Increased numbers of 3 ratings would ensure that specific policy actions are identified to address the concept, and of 4 ratings that these policy actions are taken seriously through an intention to monitor the concept.

The recent focus on the importance of mainstreaming disability in the discussion on the millennium development goals (MDGs) reflects, firstly, the lack of consideration of disability within the MDGs generally, and secondly, should be an impetus for ensuring that the needs of disabled people and other vulnerable groups are made more explicit in policies.

One of the aims of the AU is to ‘mainstream gender in all programmes and activities of the union.’ (AU n.d.). Alongside the mainstreaming of gender, there needs to be mainstreaming of disability needs and those of other vulnerable groups in all the policies and related documentation. The focus should be on diversity management to address all needs of all people.

## Footnotes

1 Disability as a holistic experience is a barrier to accessing health care but disability is created by a combination of a person’s health condition and a range of barriers and facilitators in the person’s life context.

2 The full title of the EquitAble project is ‘Enabling universal and equitable access to healthcare for vulnerable people in resource poor settings in Africa’ and it is funded by the European Union Funding Programme 7 (FP7). Further information is obtainable at http://www.sintef.no/Projectweb/Equitable/.

3 The remaining 7 core concepts were mentioned very rarely or not at all.

4 When using the term ‘policy/ies’ the reference intended includes strategy documents, plans of action, and similar documents that provide guidance and set out principles for users of the documents.

## Appendix 1

**APPENDIX 1:** Example of a completed matrix analysis.

Policy Title: Africa health strategy: 2007−2015. AU document, April 2007.														

Concepts	Number of mentions	Limited resources	Increase RR of morbidity	MCM	WHH	Children (with special needs)	Aged	Youth	Ethnic minorities	Displaced populations	Living away from services	Suffering from chronic illness	Disabled	Universal
Protection from harm	1	0	0	2	0	0	0	0	0	0	0	0	0	1
Prevention	5	0	0	1	0	0	0	1	0	1	0	0	0	1
Autonomy	0	0	0	0	0	0	0	0	0	0	0	0	0	0
Privacy	0	0	0	0	0	0	0	0	0	0	0	0	0	0
Participation	5	0	0	0	0	0	0	0	0	0	0	0	0	3	
Liberty	-	0	0	0	0	0	0	0	0	0	0	0	0
Non-discrimi-nation	8	0	0	2	0	0	0	0	0	0	0	0	0	2
Cultural res-ponsiveness	5	0	0	0	0	0	0	0	0	0	0	0	0	1
Family resources	0	0	0	0	0	0	0	0	0	0	0	0	0	0
Family Support	0	0	0	0	0	0	0	0	0	0	0	0	0	0
Integration	1	0	0	0	0	0	0	-	0	1	0	0	0	1
Contribution	0	0	0	0	0	0	0	0	0	0	0	0	0	0
Coordination of services	18	0	0	0	0	0	0	0	0	0	0	0	0	18
Capacity building	20	0	0	2	0	0	0	0	0	0	0	0	0	3
Capability based services	0	0	0	0	0	0	0	0	0	0	0	0	0	0
Individualised services	4	0	0	0	0	0	0	0	0	1	0	0	0	1
Account-ability	9	0	0	0	0	0	0	0	0	0	0	0	0	3
Quality	7	0	0	0	0	0	0	0	0	0	0	0	0	3
Access	8	0	0	0	0	0	0	0	0	0	0	0	0	3
Efficiency	8	0	0	0	0	0	0	0	0	2	0	0	0	2
Number of times VG mentioned	-	0	0	6	0	0	1	1	0	4	0	0	1	78

WHH, women headed households; MCM, mother/child mortality; RR, relative risk.

## Appendix 2

**APPENDIX 2:** Summary analysis of selected vulnerable groups and core concepts in 11 regional policies.

Policy	LR	WHH	Children with special needs	Elderly	Youth	Ethnic mino-rities	Displaced pop	Living far from services	With chronic illness	With disabilities	Universal
1. Abuja declaration on HIV/AIDS	-	-	Prevention and amelioration - 1^§^	-	Prevention and amelioration −1 Prevention and amelioration – 2	-	-	-	-	-	Anti-discrimination – 1 Service coordination and collaboration – 3 Prevention and amelioration – 1 Prof and system capacity building – 2 Access – 3 Quality – 1
2. African charter on the rights and welfare of the child – 1990	-	-	Anti-discrimination-1 services – 2 Access – 1	-	-	-	-	-	-	-	Anti-discrimination – 2 Individualised appropriate services – 2 Access – 1
3. Protocol to the African charter on human and people’s rights on the rights of women in Africa – 1995, adopted in 2003	-	-	-	Individualised appropriate services – 1	-	-	-	Individualised appropriate services – 1	-	Individualised appropriate services – 1	Prevention and amelioration – 1 Individualised appropriate services – 1 Access – 1
4. African charter on human and people’s rights – 1981	-	-	-	Protection from harm – 1 Prevention & amelioration- 1	-	-	-	-	-	Protection from harm – 1 Prevention and amelioration – 1	Protection from harm – 1 Prevention and amelioration – 1
5. Africa summit on roll back Malaria – 25 April 2000	-	-	Protect from harm – 1	-	Protection from harm – 1 Prof and systems capacity building – 2 Efficiency – 1	-	-	Efficiency – 3	-	-	Protection from harm – 1 Anti-discrimination – 1 Service coordination and collaboration – 3 Prof and systems and capacity building – 2 Quality – 1 Access – 3 Efficiency – 3
6. Pan-African forum for children – OAU document – May 2001			Prof and systems capacity building – 2 Individualised appropriate services – 1		Protection from harm – 2 Prevention and amelioration – 3 Anti-discrimination – 3 Prof and systems capacity building – 2 Individualised appropriate services – 2 Quality – 1		Prof and systems capacity building – 2	-	-	-	Access – 2
7. Maputo declaration on HIV/AIDS, Tuberculosis, Malaria and other related infectious diseases – July 2003	-	-	-	-	-	-	-	-	Service coordination and collaboration – 2 Prof and system capacity building – 2 Access – 1	-	Prevention and amelioration – 1 Service coordination and collaboration – 1 Prof and system capacity building – 1
8. Draft Continental policy framework for the promotion of sexual and reproductive health rights – AU October 2005	-	-	-	Individualised appropriate services – 1	Prevention and amelioration – 1 Cultural responsiveness – 1 Service coordination and collaboration – 1 Individualised appropriate services – 1	-	-	-	Prevention and amelioration – 1 Service coordination and collaboration – 1 Individualised appropriate services – 1	Individualised appropriate services – 1	Protection from harm – 3 Prevention and amelioration – 1 Anti-discrimination – 2 Cultural responsiveness – 1 Service coordination and collaboration – 1 Prof and system capacity building – 2 Individualised appropriate services – 1 Accountability – 1 Quality – 3 Access – 1
9. Plan of action on sexual and reproductive health and rights (Maputo Plan of Action) 2007–2010	-	-	-	-	Individualised appropriate services – 1	-	-	-	Service coordination and collaboration – 1 Access – 1	-	Prevention and amelioration – 1 Anti-discrimination – 2 Cultural responsiveness – 1 Service coordination and collaboration – 1 Prof and system capacity building – 3 Individualised appropriate services – 1 Accountability – 2 Quality – 1 Access – 2
10. AU Youth Charter – AU July 2006	-	-	-	-	Protection from harm – 5 prevention and amelioration – 4 Anti-discrimination-4 Access – 1	-	-	Access–1	-	Anti-discrimination – 2 Access – 1	-
11. Africa health strategy: 2007–2015	-	-	-	-	Prevention and amelioration – 1	-	Prevention and amelioration – 1 Individualised appropriate services -2	-	-	-	Protection from harm – 1 Prevention and amelioration – 2 Anti-discrimination – 4 Cultural responsiveness – 5 Service coordination and collaboration – 18 Prof and system capacity building – 20 Individualised appropriate services – 2 Accountability – 9 Quality – 7 Access – 8 Efficiency – 8

OAU, Organisation for African Unity; AU, African Union; LR, limited resources; WHH, women headed households.
